# Gradient descent–based linear regression: A novel framework for Kayseri Organized Industrial Zone wastewater treatment effluent parameters estimation

**DOI:** 10.1002/jeq2.70204

**Published:** 2026-06-12

**Authors:** Mahmut Sami Sasmazturk, Alper Solmaz, Talip Turna

**Affiliations:** ^1^ Department of Management Information Systems‐Faculty of Administrations and Management Sciences Iskenderun Technical University Hatay Turkey; ^2^ Department of Environmental Protection and Control‐Iskenderun Vocational School of Higher Education Iskenderun Technical University Hatay Turkey; ^3^ Department of Parks and Garden Plants‐Diyarbakır Vocational School of Higher Education Dicle University Diyarbakır Turkey

## Abstract

In this study, a method is proposed to develop a model for the estimation of chemical oxygen demand (effluent chemical oxygen demand [e‐COD]), suspended solids (effluent suspended solids [e‐SS]), and pH (effluent pH [e‐pH]) parameters from the discharge water parameters using various parameters of the wastewater treatment plant of the Kayseri Organized Industrial Zone (KOIZ‐WWTP). A gradient descent algorithm (GDA) based on machine learning, which is widely used to detect linear regression parameters, is proposed. In the first stage of the two‐stage study, the relationships between each parameter were determined, and correlational selection was applied to the input parameters accordingly. Thus, the relationships of 11 input parameters with the target parameters were determined and the parameters with high correlation between them were determined and selected. A median filter was applied to all datasets used, thus smoothing out potential outliers and reducing noise. In the second stage the batch gradient descent variant of the GDA machine learning algorithm was used to determine model parameter values. The model was applied to the training dataset and its accuracies were obtained on the test dataset. Root mean square error (e‐COD: 0.2771, e‐SS: 0.2792, e‐pH: 0.3240), variance accounting factor (e‐COD: 91.9362, e‐SS: 92.2587, e‐pH: 88.6390), and *R* ^2^_adj_ (e‐COD: 0.910, e‐SS: 0.913, e‐pH: 0.873) were obtained as performance metrics. Thanks to this study, the model parameters were determined fairly accurately iteratively without overfitting. With this study, it is planned to reduce the labor force of this and similar facilities in terms of consumables, equipment, and most importantly time, by accurately estimating the effluent parameters of KOIZ‐WWTP. Furthermore, this framework directly contributes to environmental sustainability and operational efficiency by offering potential energy savings in aeration processes and reducing the dependency on extensive laboratory analyses.

## Plain Language Summary

A novel gradient descent‐based linear regression framework was developed to estimate effluent parameters (effluent chemical oxygen demand [e‐COD], effluent suspended solids [e‐SS], and effluent pH [e‐pH]) in the wastewater treatment plant of the Kayseri Organized Industrial Zone. Correlation‐based feature selection and median filtering were applied to enhance model robustness and reduce noise from operational anomalies. The batch gradient descent algorithm was used to iteratively optimize regression coefficients, yielding high predictive accuracy without overfitting. Strong correlations were identified between influent and biological treatment parameters, especially biological treatment–mixed liquor suspended solids, which showed dominant influence across all output variables. The model achieved high performance metrics on test data: Root mean square error (e‐COD: 0.2771, e‐SS: 0.2792, e‐pH: 0.3240), variance accounting factor (VAF) (>88%), and *R* ^2^adj (>0.87). The proposed approach offers a cost‐effective alternative to sensor‐based monitoring, reducing labor, maintenance, and analysis time in wastewater operations.

**Abbreviations**
ANN: artificial neural network
B‐MLSS: biological treatment—mixed liquor suspended solids
BOD: biochemical oxygen demand
B‐pH: biological treatment—aeration basin pH
B‐SM: biological treatment—settleable solids
B‐T: biological treatment—temperature
COD: chemical oxygen demand
e‐COD: effluent chemical oxygen demand
e‐pH: effluent pH
e‐SS: effluent suspended solids
GDA: gradient descent algorithm
i‐COD: influent chemical oxygen demand
i‐pH: influent pH
i‐SS: influent suspended solids
i‐TN: influent total nitrogen
i‐TP: influent total phosphorus
KOIZ: Kayseri Organized Industrial Zone
ML: machine learning
MSE: mean square error
OLS: ordinary least squares
RMSE: root mean square error
SM: settleable
SS: suspended solids
TN/Top‐N: total nitrogen
TP/Top‐P: total phosphorus
TSS: total suspended solids
VAF: variance accounting factor
WWTP: wastewater treatment plant

## 1 INTRODUCTION

Digitalization has not only maximized the economic development of industries but also positively contributed to environmental pollution monitoring and prioritization (Wen et al., 2021). In environmental protection applications, digitalization continues to be utilized in many areas such as air quality (Bashir Shaban et al., 2016), characterization of soil pollutants (Teng et al., 2014), drinking and utility water quality (Noori et al., 2019), determination of pollutants in large water bodies such as seas and oceans (F. Wang et al., 2020), and identification and modeling of macro and micro pollutants in wastewater (Zolkefli et al., 2020). As in many branches of science, various modeling and prediction methods have been used in water and wastewater treatment. These methods attempt to model and predict data that is difficult to establish numerical relationships with each other. These machine learning (ML)‐based methods allow us to establish relationships between data with high accuracy, enabling us to establish relationships between previously uncorrelated data (Shao et al., 2023; Wan et al., 2022; Z. Ye et al., 2020). Recent studies highlight that while managing complex and nonlinear data streams in modern wastewater treatment remains challenging, ML approaches offer unprecedented opportunities for optimizing operational sustainability (Khasnabis et al., 2025; Vanrolleghem et al., 2025).

ML algorithms, a subbranch of artificial intelligence, attempt to mimic human intelligence by processing complex and excessively complex data with no established relationships. In this context, the data are organized and classified, and the target output parameter is estimated using the available data (Alpaydin, 2020; Géron, 2019; Lecun et al., 2015; Vanrolleghem et al., 2025; Z. Ye et al., 2020). This imitation ability has been used in real‐world applications in environmental systems, but the large amount and variability of available data has led to the inadequacy of classical software, leading to increased interest in artificial neural networks (ANNs), which are models that increase data‐driven processing power (Chen et al., 2020). In ANNs, each neuron in a fully interconnected neural network contains learnable parameters (weights and biases) and functions as a universal function estimator (Yegnanarayana, 2009).

The random forest technique, a statistical learning method developed by Breiman, creates a decision tree from data. Estimation of the chemical oxygen demand (COD), total phosphorus (TP), and total nitrogen (TN) parameters has been done in the literature (Cheng et al., 2023; F. Wang, Wang, et al., 2021). Support vector regression, like ML, attempts to establish relationships between input and output parameters to explain nonlinear relationships. Parameters such as pH, total suspended solids (TSS), biochemical oxygen demand (BOD), and COD have been estimated in the literature (Jana et al., 2022). In another study, parameters such as BOD, COD, PO_4_, and NO_3_ were estimated by modeling with deep neural network (Jafar et al., 2022). On the other hand, a Gaussian process model was used in a different study. This model, which maps inlet and outlet parameters, was developed to estimate the effluent parameters of a full‐scale wastewater treatment plant (WWTP) (Hvala & Kocijan, 2020).

Gradient descent algorithm (GDA) is one of the algorithms that try to explain the relationships between the parameters by establishing correlations between the parameters that are very numerous in a business and whose relationship with each other cannot be determined numerically. It exists in various variants, including batch gradient descent, stochastic gradient descent, and mini‐batch gradient descent (Haji & Abdulazeez, 2021; Ruder, 2016; X. Wang, Yan, et al., 2021). Batch gradient descent processes all data at once, while stochastic gradient descent uses a single sample at a time, while mini‐batch gradient descent takes the middle ground with small datasets. GDA attempts to optimize parameters across a large amount of data, determining weights between parameters to determine their relationships. This reduces the number of input/output parameters and, consequently, reduces the cost of measuring those parameters.

WWTPs are professionally designed and constructed to meet effluent standards. Furthermore, optimizing operating costs is crucial today, given the high cost of energy. Models (such as activated sludge model) have been developed that optimize various parameters such as temperature, pH, alkalinity, and TSS to optimize operating costs (Khasnabis et al., 2025; Pisa et al., 2019; Salgot & Folch, 2018). However, due to constant oscillations in flow and pollution loads in treatment plants, the number of variable parameters is quite high, and these models cannot account for many variables, resulting in a low performance/cost ratio (Borzooei et al., 2019; D. Wang, Thunéll, et al., 2021).

To deliver high‐quality treated wastewater to the aquatic environment, a WWTP must be operated professionally. Professional operation requires daily, and sometimes hourly, analyses across multiple units within the plant (influent, sedimentation, biological treatment, sludge disposal, etc.). These analyses take a certain amount of time, but this also delays clear decision‐making during plant operation. To overcome this problem, online sensors have been developed using technology and are being used in areas such as biological treatment, sludge removal, and energy consumption (Haimi et al., 2013; Nawaz et al., 2022; Zhang et al., 2020). Thanks to the numerous online sensors (BOD, COD, nitrogen, etc.), better quality effluent can be achieved in treatment plants. However, when operating in WWTPs with numerous pollutants, these sensors cannot consistently and consistently provide accurate results due to biofilm, particles, or harsh chemical conditions, and therefore may require maintenance, repair, and replacement (Ching et al., 2021). Many mathematical models are being studied to reduce the dependence of facilities on these and similar sensors and to determine their performance (Ching et al., 2021; Guo et al., 2015; R. Huang et al., 2021; Lee et al., 2022). In addition to these models, correlating facility operating parameters with artificial intelligence techniques will increase the performance of these facilities (Ching et al., 2021; Sweeney & Kabouris, 2020). Furthermore, this correlation will reduce the sampling required for facility operation, thereby reducing analysis costs. Furthermore, through optimization, energy consumption will also be reduced (Bagherzadeh et al., 2021; D. Wang, Thunéll, et al., 2021).

In this study, a modeling study was conducted specifically for wastewater treatment plant of the Kayseri Organized Industrial Zone (KOIZ‐WWTP) to minimize such problems experienced in treatment plants. GDA was designed as a ML algorithm to determine the parameter values for the proposed linear regression model. Correlations were determined between the plant's flow rate (*Q*); inlet pH (influent pH [i‐pH]); suspended solids(SS) (influent suspended solids [i‐SS]); settleable solids (influent settleable solids); TN (influent total nitrogen [i‐TN]); TP (influent total phosphorus [i‐TP]); COD (influent chemical oxygen demand [i‐COD]); and the aeration basin pH (biological treatment—aeration basin pH [B‐pH]), temperature (biological treatment—temperature [B‐T]), mixed liquor suspended solids (biological treatment—mixed liquor suspended solids [B‐MLSS]), and settleable solids (biological treatment—settleable solids [B‐SM]) parameters in the biological treatment unit, and their weights were calculated. A significant reduction in the number of parameters was achieved by selecting variables. Thus, the estimation of output variables with sufficient and necessary input variables was made more consistently. Median filtering was applied to the dataset to smooth out potential outliers and reduce noise. According to the determined parameters, the output variables of the effluent, effluent chemical oxygen demand (e‐COD), effluent suspended solids (e‐SS), and effluent pH (e‐pH) values, were estimated. Although complex nonlinear models such as ANNs are prevalent in literature, this study proposes a linear regression framework to prioritize model interpretability and computational efficiency for real‐time plant operation. The high correlation structure observed in the preliminary analysis suggests that a robustly optimized linear model is sufficient for this specific application, offering a transparent “white‐box” solution that plant operators can easily interpret compared to “black‐box” deep learning architectures.

## 2 MATERIALS AND METHODOLOGY

### 2.1 KOIZ‐WWTP description

**FIGURE 1:**
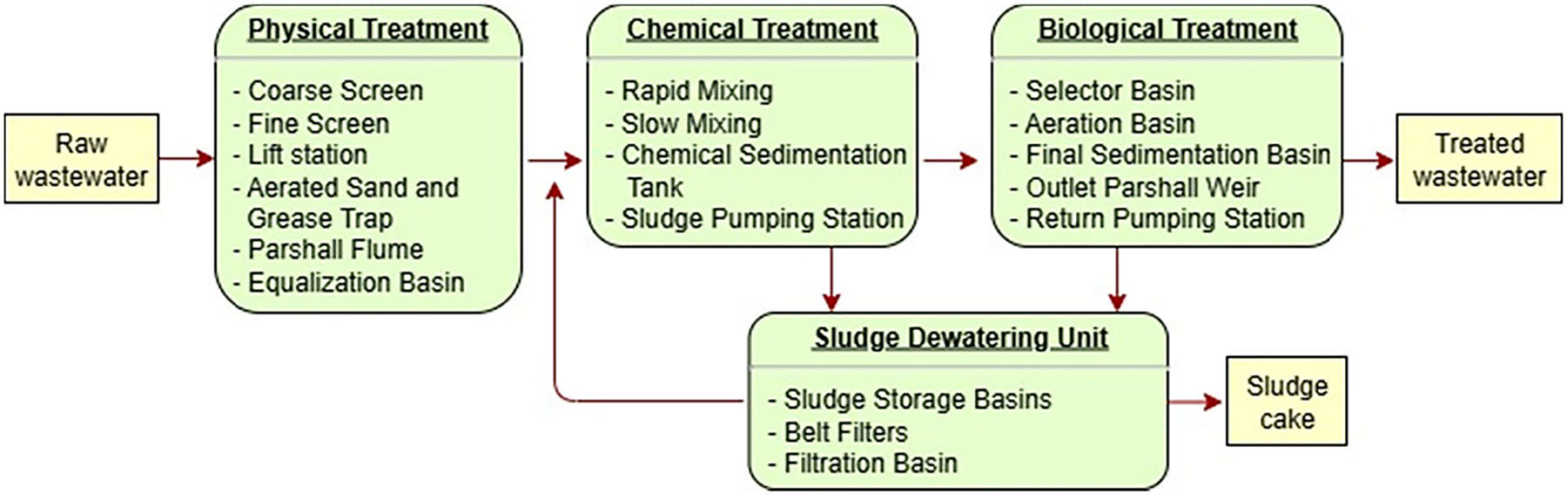
Wastewater treatment plant of the Kayseri Organized Industrial Zone (KOIZ‐WWTP) schematic diagram.

This region, designated as a KOIZ‐WWTP (Kayseri, Turkey), hosts over 1200 factories. The region's production consists of approximately 26.8% household goods, electrical appliances, and metal products; 22.9% furniture and wood products; 10.2% packaging, plastics, and recycling; 10.1% construction materials; 9.8% textiles; and 20.2% machinery, food, electrical‐electronics, and paint‐chemicals. In terms of wastewater generation, 27.4% comes from paper (recycling), printing, and advertising; 25.9% textiles; 11.5% paint, chemicals, coatings, and cleaning products; 10.1% packaging, plastics, and recycling; 9.4% food; and the remaining 15.7% comes from other sectors. A schematic diagram of the KOIZ‐WWTP is presented in Figure 1. The processes, which includes physical, chemical, biological and sludge dewatering units, was designed for a flow rate of 60,000 m^3^/day, while physical, chemical, sludge dewatering, and other auxiliary facilities (chemistry, blower, transformer, and administrative building) were constructed for a flow rate of 60,000 m^3^/day, and the biological treatment unit was constructed for a flow rate of 400,000 m^3^/day.

### 2.2 Methodology

**FIGURE 2:**
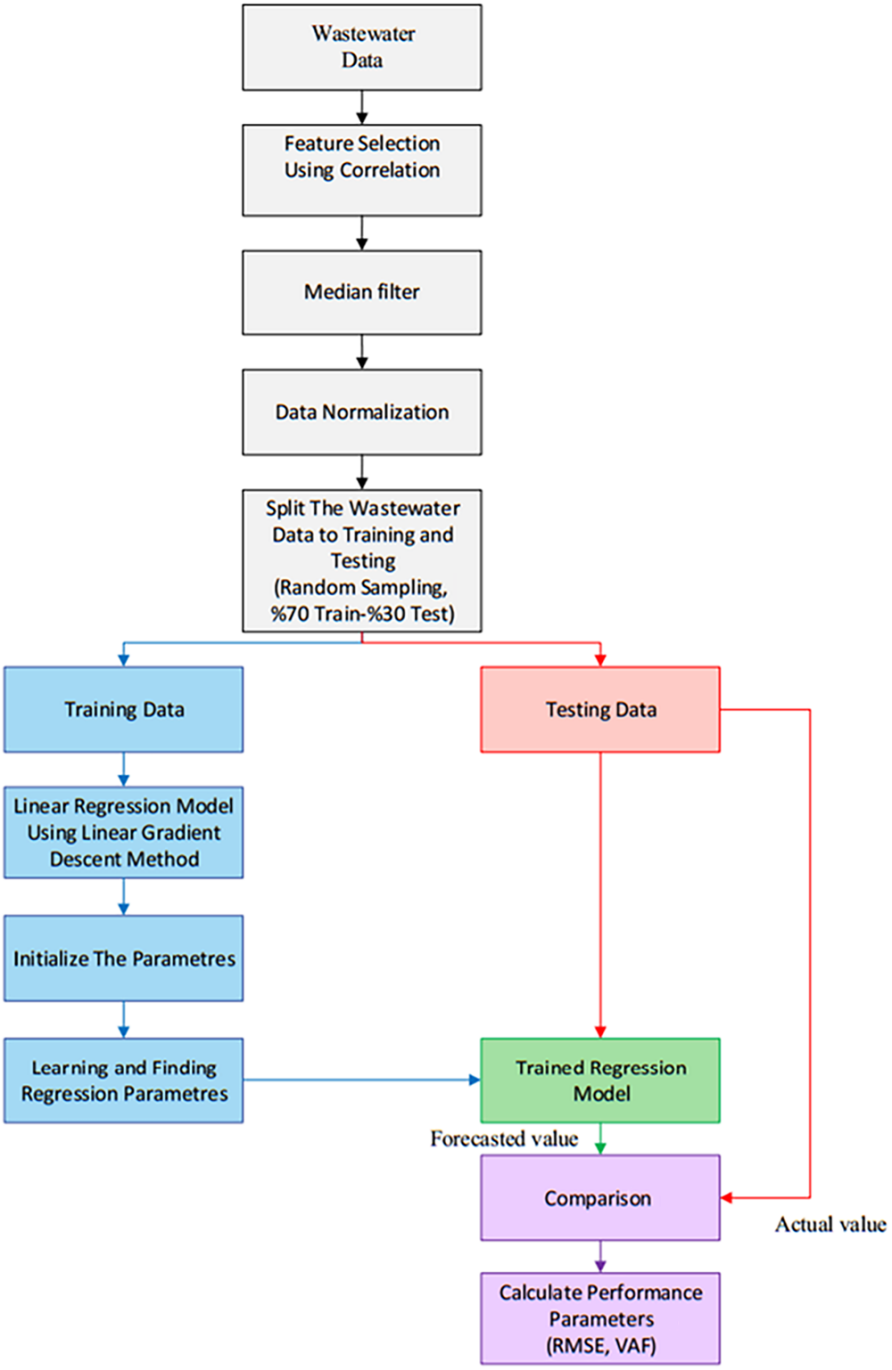
Proposed framework for predictions of effluent parameters.

This study aimed to integrate parametric data related to wastewater into a regression‐based prediction model. The flowchart shown in Figure 2 describes the systematic process, which includes data preprocessing, model training, and performance evaluation. In the first stage, observational data related to wastewater were collected.

Feature selection was performed using correlation analysis to determine the relationship between independent variables (Taylor, 1990). This step eliminated unnecessary variables or variables with low information contribution from the model. A median filter was applied to the dataset to smooth out potential outliers and reduce noise (Martin & Lutz, 1979; Pratt, 2013; Tukey, 1977). Then, a normalization process was performed to eliminate scale differences between variables. This process supports robust optimization during the model's learning process. The processed dataset was randomly divided into 70% training and 30% test data (Makwana et al., 2023; Martino et al., 2018). This distinction is important for evaluating the model's generalization ability. A linear regression model was developed using randomly separated training data. Parameter updates and the determination of optimal model parameters were performed using a linear GDA (Ruder, 2016). This ML algorithm enables the model to learn its parameters by minimizing the error function.

After the training process is completed, optimal model parameters are determined using the training dataset, and test data are applied to the model; the predicted values are compared with actual observations. Model accuracy is assessed using two fundamental metrics widely used in the literature: root mean square error (RMSE): This represents the average magnitude of the prediction error. Variance accounting factor (VAF): This measures the explanatory power of the model. Additionally, the performance metric of the adjusted multiple correlation coefficient (*R* ^2^_adj_) was also used to determine model accuracy (Plevris et al., 2022).

#### 2.2.1 Data

Data were obtained from the physical treatment plant effluent (which we refer to as raw wastewater) (chemical treatment inlet), the biological treatment plant, and the final sedimentation tank effluent, where reliable analyses can be performed. A total of 2558 datasets were used for the 6‐year period, from January 1, 2015, to December 31, 2021. “Raw wastewater” samples were taken after the exit of the grit trap unit of the facility, samples from the “Biological treatment” section were taken from the aeration pool, and the “Effluent” section was taken from the final sedimentation pool exit point, that is, before discharge into the receiving environment. In this study, 70% (1720) of the total dataset was allocated for the training phase, while the remaining 30% (737) was reserved for testing. Test data were selected randomly. The 11 input parameters are as follows: (1) flow rate, (2) pH, (3) SS, (4) settleable matter (SM) (60 min), (5) top‐N, (6) top‐P, (7) COD. Biological treatment (aeration tank) parameters are (8) B‐pH, (9) B‐temperature, (10) mixed liquor suspended solids (B‐SS), and (11) B‐SM (30 min). The three estimated output parameters are (1) e‐pH, (2) e‐SS, and (3) e‐COD parameters at the final sedimentation outlet of the facility.

#### 2.2.2 Data Preprocessing

The data used in the study were transferred to the modeling process by applying appropriate data preprocessing steps. First, the correlation relationship between these independent variables presented in Table 1 and the dependent variables pH, SS, TN, TP, and COD was calculated using Equation (1).

**TABLE 1:** 84 months of measured data of water quality parameters for Wastewater treatment plant of the Kayseri Organized Industrial Zone (KOIZ‐WWTP) process.

Location	Parameter	Unit	Min	Mean (*μ*)	Max	SD (*σ*)	*μ* ± *σ*		*μ* ± 2*σ*	
							*μ* + *σ*	*μ* − *σ*	*μ* + 2*σ*	*μ* −2*σ*
Raw wastewater	Flow rate	m^3^/day	1850	24,810	42,500	5086	19,724	29,897	14,637	34,984
	pH	–	6.42	7.33	8.33	0.28	7.05	7.62	6.76	7.90
	SS	mg//L	480.0	1302.64	7910	340.76	961.88	1643.40	621.11	1984.16
	SM (60 min)	mL/L	7.00	46.89	160.00	16.31	30.58	63.19	14.28	79.50
	TN	mg//L	12.00	31.41	71.80	8.10	23.31	39.51	15.21	47.61
	TP	mg//L	2.05	8.10	28.70	3.63	4.47	11.74	0.83	15.37
	COD	mg//L	135.00	1452.72	2481.00	323.47	1129.25	1776.19	805.78	2099.67
Biological treatment	pH	–	6.69	7.48	8.14	0.18	7.30	7.66	7.12	7.84
	Temperature	^°^C	11.05	22.19	28.35	3.48	18.72	25.67	15.24	29.15
	SS	mg/L	1164.00	2699.53	5608.00	796.71	1902.82	3496.24	1106.10	4292.96
	SM (30 min)	mL/L	42.00	263.51	990.00	140.37	123.14	403.89	–	544.26
Effluent	pH	–	6.38	7.58	8.29	0.21	7.37	7.78	7.16	7.99
	SS	mg/L	3.00	20.43	73.00	8.00	12.42	28.43	4.42	36.43
	COD	mg/L	14.40	55.60	180.00	19.31	36.29	74.91	16.99	94.21

Abbreviations: COD, chemical oxygen demand; SD, standard deviation; SM, settleable matter; SS, suspended solids; TN, total nitrogen; TP, total phosphorus.

This table provides a statistical summary of various parameters for raw wastewater, the biological treatment process, and the treated (effluent) water. Parameters include flow rate, pH, SS, TN, TP, COD, and temperature. Minimum, maximum, mean (*μ*), standard deviation (*σ*), and specific standard deviation ranges (*μ* ± *σ*, *μ* ± 2*σ*) are given in the table. The data in this table indicate significant changes in the physicochemical properties of raw wastewater after passing through the biological treatment process. Significant improvements are observed, particularly in the pH, SS, TN, TP, and COD parameters. The standard deviation (*σ*) values reflect the variability of the process. The biological treatment process improves the composition of the wastewater, making it more compliant with environmental standards.

The obtained correlation coefficients play a significant role in determining the degree of interaction between the dependent and independent variables (Asuero et al., 2006). Accordingly, the coefficient values were analyzed to select the most suitable variables affecting the predictability of the dependent variables. During the variable selection process, the optimal set of independent variables was determined for each dependent variable based on the magnitude and statistical significance of the correlation coefficients, and these variables were used in the modeling phase. This method aims to increase model accuracy and minimize the margin of error resulting from excessive parameter usage.

(1) $$r=\frac{\sum_{t=1}^{n}\left(X_{t}-\bar{X}\right)\left(Y_{t}-\bar{Y}\right)}{\sqrt{\sum_{t=1}^{n}{\left(X_{t}-\bar{X}\right)}^{2}}\sqrt{\sum_{t=1}^{n}{\left(Y_{t}-\bar{Y}\right)}^{2}}}$$

In the later stages of the data preprocessing process, median filtering was applied to increase the dataset's stability and reduce noise. The median filter not only reduces noise and maintains statistical robustness, but also provides a reliable filtration mechanism by minimizing the impact of outliers in the dataset. In particular, an examination of the wastewater dataset revealed that potential anomalies that may occur during operational processes lead to measurement errors. Because such errors can negatively impact the dataset's accuracy and analysis results, a filtering method that maintains data reliability is necessary.

**FIGURE 3:**
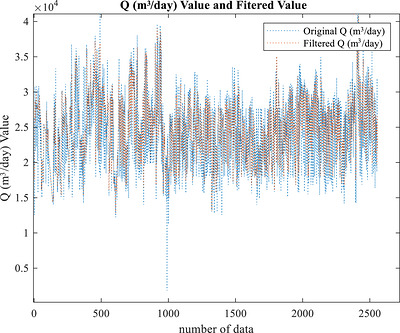
Data preprocessing: Median filtering effect of input *Q* (m^3^/day) variable.

In this context, the use of the median filter was considered a suitable approach for eliminating impulsive noise, limiting the impact of anomalous measurement points, and preserving data integrity. Compared to traditional linear filters, the median filter is specifically preferred for its “edge‐preserving” capability. This characteristic makes it highly effective at suppressing impulse noise caused by operational anomalies or temporary sensor malfunctions without blurring the underlying process dynamics. Consequently, the median filter was applied as an effective and reliable filtration technique to eliminate measurement errors in the wastewater dataset and to facilitate more reliable data use in analytical processes. Figure 3 presents a sample dataset obtained before and after applying the median filtering process.

This figure provides a comparative analysis of the effects of the filtering process on the data. This transformation, shown in the figure, demonstrates the effectiveness of the median filter in removing noise, providing data stabilization, and increasing statistical reliability. In this context, the application of the median filter is considered to support more robust results in modeling and forecasting processes (Gabbouj et al., 1992).

In addition to noise reduction, strict protocols were applied to handle data gaps and potential sensor calibration errors inherent in the 6‐year operational period. Instead of using imputation techniques that could introduce synthetic bias, a “complete case analysis” approach was adopted. Records containing missing values or clear sensor malfunctions were excluded from the dataset. Consequently, the study utilized only the 2558 fully validated data points where all 11 input parameters and output values were available, ensuring that the model training was based on high‐fidelity ground truth data.

Data normalization is a critical preprocessing method that eliminates scale differences in the dataset, making it suitable for the modeling process. This process plays a crucial role in improving model performance, ensuring the reliability of statistical comparisons, and minimizing the impact of anomalous values. Examining the dataset presented in Table 2, it is observed that there are significant scale differences between the variables. In particular, the min–max values and mean values are quite different from each other in terms of the measurement ranges of the variables. This makes it difficult for ML models to evaluate variables in a balanced manner, which can reduce prediction accuracy.

**TABLE 2:** Random sampling psudocode.

*FUNCTION randomSampling* (data,trainNumber): # Determine the number of data points data_n = size of *data* (number of rows) # Generate a random permutation of indices tmp = randomly permute indices from 1 to data_n # Select the first ‘trainNumber’ indices for the training set index_train = first ‘trainNumber’ elements of tmp train = data at index_train # Assign remaining indices to the verification set index_verif = remaining elements of tmp verif = data at index_verif *RETURN* train,verif	

In this context, the *Z*‐score normalization method, expressed in Equation (2), was applied to eliminate scale discrepancies in the dataset. *Z*‐score normalization converts data points to a standard scale by calculating the degree of deviation of each variable from its mean and contributes to a more robust modeling process by balancing the differences between variables (L. Huang, 2022). *Z*‐score normalization was considered an essential method and was implemented within the scope of the study to eliminate scale differences in the dataset, increase the model's learning capacity, and ensure the accuracy of statistical analyses.

(2) $$x_{ij}^{\prime }=\frac{x_{ij}-\mu_{i}}{\sigma_{i}}$$

Here $x_{ij}$ is the c measurement of ith sensor, $\mu_{i}$ is the mean of ith sensor measurements, $\sigma_{i}$ is the corresponding standard deviation, and $x_{ij}^{\prime }$ is the normalized value of the jth measurement of ith sensor. This normalization approach mitigates the impact of varying measurement units and enhances comparability in subsequent analyses. The dataset was randomly divided into training and test sets to assess the model's generalization ability. This process prevents systematic deviations in the data distribution, allowing for consistent analysis of the model's performance across different data samples. In this process, the specified data separation was achieved using random sampling. Random sampling increases the representativeness of the training and test sets by randomly selecting data points within a specific probability distribution (Hastie et al., 2005). Thus, the knowledge acquired by the model during training can be tested in accordance with real‐world data, allowing for more accurate assessments of accuracy and reliability. The applied random sampling method was chosen to reduce the risk of overfitting (overfitting) and increase generalization success by preserving the statistical integrity of the dataset. This data splitting method, used in the modeling phase, supports both the efficient progress of the training process and the achievement of more reliable results during the testing phase. The random sampling method is expressed as a pseudocode. This pseudocode represents the random sampling method used to separate the dataset into training and test sets. The data are first shuffled randomly to prevent systematic sorting errors and then divided according to the specified training ratio to achieve balanced data separation.

#### 2.2.3 Linear regression‐based model training and parameter optimization

In this study, linear regression was used to linearly model the relationship between the dependent variable and the explanatory variables. The regression model is based on certain assumptions: the assumption of a linear relationship between the dependent and independent variables, normal distribution of errors with mean zero, constancy of variances (homoscedasticity), and independence of observations. The basic mathematical expression of the model is expressed in Equation (4). In Equation (5), where *β* represents the parameter vector that the model is responsible for learning, and *ε* represents the error vector, the model is expressed in vector form.

(4) $$\hat{y}\_i=h\_\beta \left(x\_i\right)=\beta_{0}+\beta_{1}x\_i_{1}+\beta_{2}x\_i_{2}+\cdots +\beta_{n}x\_in+\varepsilon \_i$$

(5) $$\hat{y}=X\beta +\varepsilon$$

While closed‐form solutions like ordinary least squares (OLS) are traditionally preferred for linear regression due to their computational efficiency on static datasets, the GDA was specifically adopted in this framework. This choice was driven by the algorithm's iterative nature, which allows for computationally efficient updates suitable for handling continuous data streams from online sensors and offers superior scalability for long‐term industrial monitoring applications compared to the matrix inversion requirements of OLS.

*Parameter updates: GDA*

Model learning is based on minimizing the loss function. The loss function is based on the mean square error (MSE) function. The loss function based on the MSE function is expressed in Equation (6). The prediction function is presented in Equation (7). Where J(β) is the cost function, m denotes the number of training examples, h_β(x_i) represents the predicted value, and y_i is the actual observed value.

(6) $$J\left(\beta \right)=\left(1\;/\;2m\right)\;\Sigma_{i}=1/m\;{\left(h\_\beta \left(x\_i\right)\;-\;y\_i\right)}^{2}$$

(7) $$h\_\beta \left(x\_i\right)=x\_i^{t}\;\beta$$

The GDA parameter update is provided by Equation (8). The derivative (gradient) equation is expressed in (9).

(8) $$\beta \_j\;≔\;\beta \_j\;-\;\alpha \;\times \;\left(\partial J\left(\beta \right)\;/\;\partial \beta \_j\right)$$

(9) $$\partial J\left(\beta \right)\;/\;\partial \beta \_j=\left(1\;/\;m\right)\;\Sigma_{i}=1^{\wedge }m\;\left(h\_\beta \left(x\_i\right)-y\_i\right)\times x\_ij$$

Here, *m* represents the sample size, *α* represents the learning coefficient, and h_β(x_i) or $\hat{y}\_i$ represents the model's estimate. The psudocode for the operation of the GDA is given in Table 3. Input: X: Feature matrix (m×n), y: Target vector (m×1), α: Learning rate (e.g., 0.01), iterations: Number of iterations (e.g., 1000), m: Number of training examples. Output: β: Parameter vector initialized to zeros (n×1).

**TABLE 3:** Gradient descent algorithm psudocode.

*FUNCTION GradientDescent* (X,y,β,α,iterations): for i from 1 to iterations: predictions←X∗β errors←predictions−y $\mathrm{gradient}\leftarrow \left(1/m\right)*\;\left(X^{t}*\mathrm{errors}\right)$ β←β−α∗gradient *RETURN* β	

Here, $X^{t}$ is the transpose of the feature matrix. $predictions(\hat{y}$): the estimated output based on current parameters, errors: how far off the predictions are from the actual target values, gradient: direction and magnitude for adjusting parameters based on total error, β: updated parameters iteratively refined to minimize prediction error. All vector and matrix operations follow linear algebra conventions.

**FIGURE 4:**
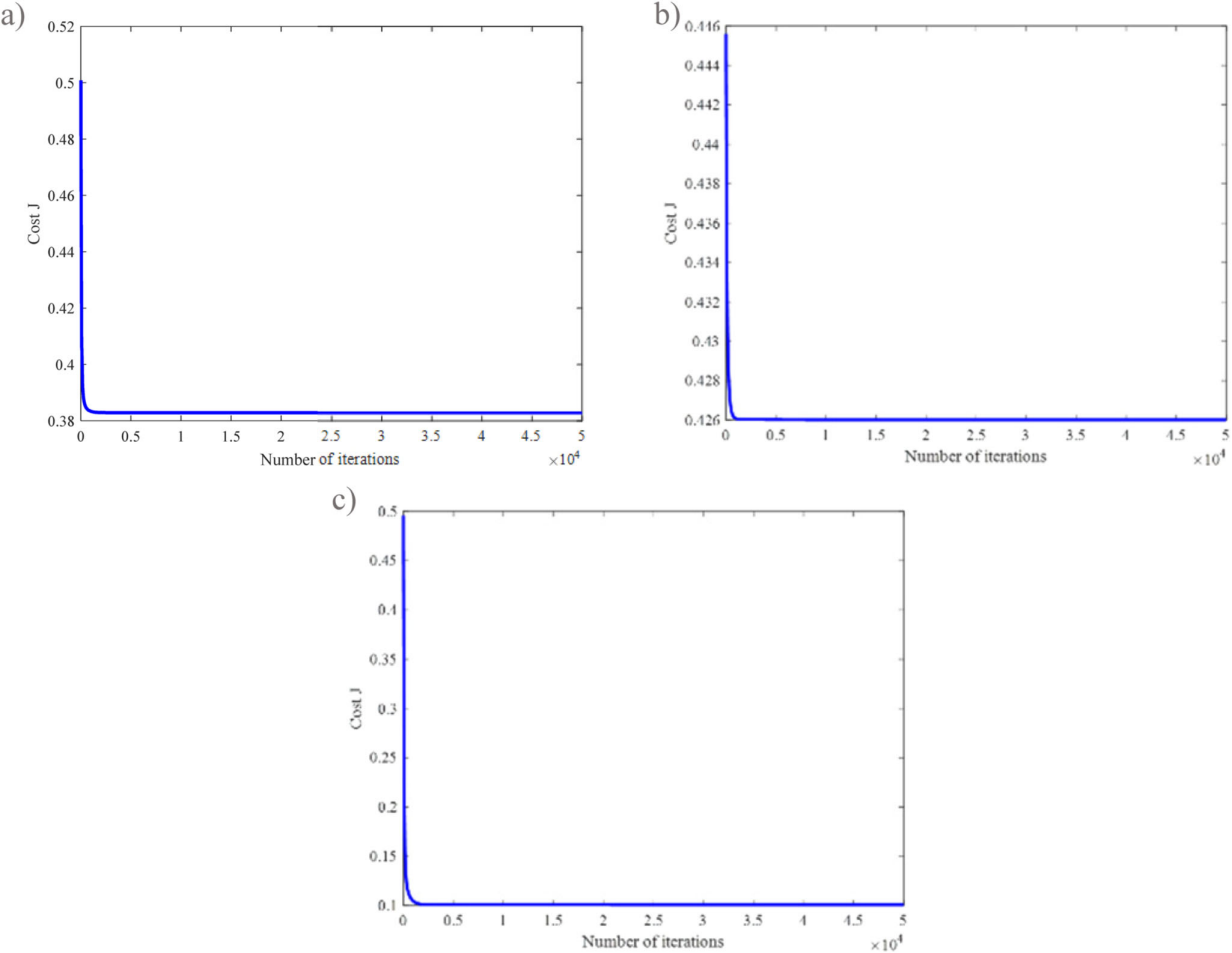
Iterative effect of the gradient descent algorithm on the cost function (*J*) in the linear regression model training process. (a) *J* function convergence graph of the effluent chemical oxygen demand (e‐COD) output, (b) *J* function convergence graph of the effluent suspended solids (e‐SS) output, and (c) *J* function convergence graph of the effluent pH (e‐pH) output.

In the implementation of the algorithm, the learning rate (α) was determined empirically through preliminary trials to ensure stable convergence without oscillation. Instead of a dynamic stopping criterion (e.g., ΔJ(β)<ε), a fixed iteration counts of 10,000 was selected as a conservative upper bound. Given the computational efficiency of the linear model on this dataset, this extended iteration counts guaranteed that the algorithm fully converged to the global minimum, as evidenced by the asymptotic behavior of the cost function curves in Figure 4.

At each iteration, parameters are updated along the gradient of the loss function, resulting in lower error values. To improve the model's generalization, the following strategies were employed: Data separation separates training and test data, reducing the risk of overfitting. Normalization, particularly in multivariate datasets, normalized features to balance the learning rate. Parameter updates were made based on the learning coefficient and error values, resulting in more balanced and faster convergence. Post‐training model validation was performed using test data to measure predictive performance ($R_{\mathrm{adj}}^{2}$, RMSE, variance accounted for [VAF]). The equations for performance metrics are presented in Equations (10), (11), and (13).

(10) $$\mathrm{RMSE}=\sqrt{\frac{1}{m}\sum_{i=1}^{m}{\left(y\_i-\hat{y}\_i\right)}^{2}}$$

(11) $$\mathrm{VAF}=1-\frac{\sum_{i=1}^{m}\left(e\_i-\bar{e}\_i\right)}{\sum_{i=1}^{m}\left(y\_i-\bar{y}\_i\right)}$$

(12) $$R^{2}=1-\frac{\sum_{i=1}^{m}{\left(y\_i-\hat{y}\_i\right)}^{2}}{\sum_{i=1}^{m}{\left(y\_\_i-\bar{y}\_i\right)}^{2}}$$

(13) $$R_{\mathrm{adj}}^{2}=1-\frac{m-1}{m-t-1}\left(1-R^{2}\right)$$

Here, e_i represents the deviation between the actual value and the estimated value. It is calculated as $e\_i=y\_i-\hat{y}\_i$. *m* represents the sample size and t represents the number of features (independent variables) in the model.

## 3 EXPERIMENTAL SETUP AND FINDINGS

### 3.1 Relationship between facility operation input parameters

It is a legal requirement for a WWTP to maintain good effluent quality. This legal requirement requires constant monitoring of the plant's operating parameters. In treatment processes using conventional activated sludge systems, the influent characteristics significantly affect the biological treatment process. Furthermore, controlling the parameters within the biological treatment process is crucial for water quality. In this context, the relationship between the influent parameters of the KOIZ WWTP and the parameters of the biological treatment unit was calculated, and the resulting correlation coefficient values are presented in Table 4.

**TABLE 4:** Pearson correlation relationship between raw wastewater influent and biological treatment monitoring parameters.

Pearson correlation	*Q*	i‐pH	i‐SS	i‐SM	i‐TN	i‐TP	i‐COD	B‐pH	B‐T	B‐MLSS	B‐SM
*Q*	1.0000	−0.1044	0.1984	0.1538	−0.0116	0.1216	0.2421	0.0479	0.0182	0.1003	−0.0079
i‐pH	−0.1044	1.0000	−0.2224	−0.2186	−0.2470	−0.0181	−0.2261	0.5639	0.0913	−0.2616	0.1068
i‐SS	0.1984	−0.2224	1.0000	0.6234	0.2498	0.2253	0.7024	0.0452	0.0936	0.3506	−0.0214
i‐SM	0.1538	−0.2186	0.6234	1.0000	0.1929	0.1221	0.6257	0.0236	−0.2600	0.3124	0.0737
i‐TN	−0.0116	−0.2470	0.2498	0.1929	1.0000	0.1354	0.2016	−0.1773	0.0042	0.4648	−0.0384
i‐TP	0.1216	−0.0181	0.2253	0.1221	0.1354	1.0000	0.2100	0.0396	0.1148	0.2127	−0.0610
i‐COD	0.2421	−0.2261	0.7024	0.6257	0.2016	0.2100	1.0000	0.1536	0.1313	0.2658	0.0226
B‐pH	0.0479	0.5639	0.0452	0.0236	−0.1773	0.0396	0.1536	1.0000	0.3015	−0.0717	0.1194
B‐T	0.0182	0.0913	0.0936	−0.2600	0.0042	0.1148	0.1313	0.3015	1.0000	0.0110	−0.0615
B‐MLSS	0.1003	−0.2616	0.3506	0.3124	0.4648	0.2127	0.2658	−0.0717	0.0110	1.0000	0.1065
B‐SM	−0.0079	0.1068	−0.0214	0.0737	−0.0384	−0.0610	0.0226	0.1194	−0.0615	0.1065	1.0000

Abbreviations: B‐MLSS, biological treatment—mixed liquor suspended solids; B‐pH, biological treatment—aeration basin pH; B‐SM, biological treatment—settleable solids; B‐T, biological treatment—temperature; i‐COD, influent chemical oxygen demand; i‐pH, influent pH; i‐SM, influent settleable solids; i‐SS, influent suspended solids; i‐TN, influent total nitrogen; i‐TP, influent total phosphorus.

When the table is examined in detail and some of the actual values are examined, it is seen that, after eliminating the dilution conditions in rainy weather, the inlet flow rate in dry weather (summer months) was 25,000–30,000 m^3^/day, while the SS value was 1000–1400 mg/L and the COD value was 1000–1500 mg/L. Thus, it can be said that there is a closer relationship between *Q* and the i‐SS and i‐COD parameters than the other parameters. For example, while the *Q* and i‐SS value was 0.1984, the *Q* was calculated as 0.2421 when looking at the i‐COD values. This may be due to the diversity of the industry and production within the organized industrial zone. Furthermore, when looking at organic pollutants among the pollution parameters, COD is one of the most important parameters. COD represents the amount of organic matter that can be oxidized under strongly acidic conditions (Raposo et al., 2008). A positive relationship is expected between COD and i‐SS. This is because an increase in the presence of solids in the environment also means an increase in i‐COD. This value, calculated as 0.7024 between the i‐SS value and the i‐COD value, is quite high compared to the relationship between other parameters. Thus, a strong positive correlation can be seen between i‐SS and i‐COD.

In the aeration tank where the biological reaction takes place, microorganisms decompose organic matter and convert it into stable end products. This system, called activated sludge, contains both organic and inorganic substances. The increase in organic matter content in the activated sludge system can be explained by the increase in the amount of microorganisms. In addition, the microorganisms contain a certain amount of C, N, P, and trace elements. In this system, where many factors such as reactor design, recycle ratio, sludge age, and hydraulic retention time interact, the parameters listed play an important role. The C:N:P ratio in these systems can generally be accepted as around 100:5:1 (Hamza et al., 2019; Metcalf & Eddy, 2013; F. Ye et al., 2011). In this context, the relationships between the B‐MLSS value and the i‐SS, i‐TP, and i‐TN parameters were calculated as 0.3506, 0.4648, and 0.2127, respectively. On the other hand, the relationship between the organic pollution (i‐COD) in the influent and the B‐MLSS value was calculated as 0.2658. Therefore, it can be said that there is a linear relationship between the influent pollution values and the B‐MLSS values, as expected. Furthermore, a neutral pH is required for optimal vital activity of the microorganisms in activated sludge. The i‐pH value, which is easily affected by production variability and seasonal changes, can easily affect the B‐MLSS value. In this context, the calculated value between the i‐pH value and the B‐MLSS value is −0.2616. This value indicates a negative linear relationship between these two parameters.

### 3.2 Relationship between facility inlet–outlet water parameters

Within the scope of the study, numerical data obtained from the relationship between some raw wastewater inlet parameters of the facility and discharge water quality parameters (e‐COD, e‐SS, and e‐pH) are presented in Table 5.

**TABLE 5:** Relationship between raw wastewater inlet parameters and discharge water parameters.

		Output		
Pearson correlation		e‐COD	e‐SS	e‐pH
Input	*Q*	−0.0580	0.0176	0.0342
	i‐pH	0.1953	0.0425	0.5111
	i‐SS	−0.1872	−0.0673	0.0005
	i‐SM	−0.1460	0.0155	−0.0364
	i‐TN	−0.3210	−0.1328	−0.2098
	i‐TP	−0.1041	−0.0301	0.0160
	i‐COD	−0.1218	−0.0425	0.0935
	B‐pH	0.1970	0.0394	0.8210
	B‐T	0.1113	−0.0959	0.2651
	B‐MLSS	−0.3182	−0.0640	−0.0904
	B‐SM	0.0586	0.0790	0.0977

Abbreviations: B‐MLSS, biological treatment—mixed liquor suspended solids; B‐pH, biological treatment—aeration basin pH; B‐SM, biological treatment—settleable solids; B‐T, biological treatment—temperature; e‐COD, effluent chemical oxygen demand; e‐pH, effluent pH; e‐SS, effluent suspended solids; i‐COD, influent chemical oxygen demand; i‐pH, influent pH; i‐SM, influent settleable solids; i‐SS, influent suspended solids; i‐TN, influent total nitrogen; i‐TP, influent total phosphorus.

When Table 5 is examined, the calculated values between the i‐pH parameter and the e‐pH and B‐pH parameters are 0.511 and 0.8210, respectively. Changes in the pH value of the plant's influent directly affect both biological treatment and the e‐pH. This relationship is easily considered positive and strong. On the other hand, the relationship between the i‐TN and e‐COD value is calculated as −0.3210. This suggests that effluent quality deteriorates in the event of a highly polluted wastewater input. Similarly, the relationship between the MLSS value in the biological reactor and the e‐COD value is −0.3182. In this case, it can be said that the change in water quality is negative when the MLSS value in the biological reactor increases or decreases.

### 3.3 Variable selection

In this study, the Pearson correlation coefficient was used as the basis for determining the independent variables to be used in the modeling process. The Pearson coefficient is a statistical measure that quantifies the direction and strength of the linear relationship between two variables. In this context, only independent variables that showed significant correlations with the target variables were included in the model, optimizing both the model's explanatory power and its predictive performance.

#### 3.3.1 Input variables for e‐COD (output COD)

The following input variables, which showed a significant correlation with the e‐COD output, were included in the model:

1. *Q*: −0.0580
2. i‐pH: 0.1953
3. i‐SS: −0.1872
4. i‐TN: −0.3210
5. i‐TP: −0.1041
6. i‐COD: −0.1218
7. B‐pH: 0.1970
8. B‐T: 0.1113
9. B‐MLSS: −0.3182

These variables exhibit low to moderate correlations with e‐COD, with parameters such as i‐TN and B‐MLSS exhibiting particularly strong negative correlations. This highlights the impact of nitrogen and B‐MLSS on e‐COD values.

#### 3.3.2 Input variables for e‐SS

The variables that show a significant relationship with e‐SS output are as follows:

1. i‐SS: −0.0673
2. i‐TN: −0.1328
3. B‐T: −0.0959
4. B‐MLSS: −0.0640

These parameters show a weak negative correlation with e‐SS. i‐TN and B‐T, in particular, stand out as important factors affecting e‐SS concentration.

#### 3.3.3 Input variables for e‐pH (output pH value)

Variables that show a significant correlation with e‐pH output are presented below:

1. i‐pH: 0.5111
2. i‐TN: −0.2098
3. i‐COD: 0.0935
4. B‐pH: 0.8210
5. B‐T: 0.2651
6. B‐MLSS: −0.0904

In particular, the B‐pH and i‐pH variables show a high positive correlation with e‐pH. This clearly demonstrates that the system's i‐pH and B‐pH values play a decisive role in e‐pH.

The variable selection based on the correlation analysis presented above ensured that the model was fed with inputs that established a significant relationship with the target variables. This approach reduced the risk of overfitting and increased the model's generalizability. Furthermore, by eliminating unnecessary variables from the model, computational costs were reduced and parameter interpretability was strengthened. In this context, correlation‐based variable selection significantly contributes to the modeling process in terms of both statistical validity and application‐oriented accuracy (Desboulets, 2018; Geweke, 1996). The results more clearly demonstrate the effects of environmental parameters on effluent water quality and enable the development of advanced prediction algorithms.

### 3.4 System modeling

Within the scope of the study, the independent parameters selected for the output variables were constructed using a linear regression model. In this model, the parameters representing the regression coefficients β were estimated using the batch GDA. This algorithm offers an iterative optimization process to minimize the cost function over the entire dataset, ensuring optimal parameter values are achieved. This method yielded the optimal parameter set that best represents the output variables of the linear regression model. These parameters, which increase the predictive power and explanatory power of the model, were calculated separately for each output variable and are presented in detail in Table 6. Thus, a robust regression structure was established in terms of both theoretical accuracy and practical validity.

**TABLE 6:** Model parameter β values of estimated effluent parameters.

Inputs	β		
	e‐COD	e‐SS	e‐pH
*Q*	0.0348	–	–
i‐pH	0.0570	–	0.0462
i‐SS	0.0025	0.0072	–
i‐SM	–	–	–
i‐TN	0.0280	0.0224	0.0246
i‐TP	0.0146	–	–
i‐COD	−0.0052	–	0.0164
B‐pH	−0.0488	–	−0.0442
B‐T	0.0450	0.0344	0.0387
B‐MLSS	−0.4012	−0.4127	−0.3965

Abbreviations: B‐MLSS, biological treatment—mixed liquor suspended solids; B‐pH, biological treatment—aeration basin pH; B‐T, biological treatment—temperature; e‐COD, effluent chemical oxygen demand; e‐pH, effluent pH; e‐SS, effluent suspended solids; i‐COD, influent chemical oxygen demand; i‐pH, influent pH; i‐SM, influent settleable solids; i‐SS, influent suspended solids; i‐TN, influent total nitrogen; i‐TP, influent total phosphorus.

Table 6 presents the parameter values (*β* coefficients) of the linear regression model estimated using the batch GDA. These coefficients, calculated separately for each output variable (e‐COD, e‐SS, and e‐pH), quantitatively reveal the impact of the relevant independent variables on the model.

The physical interpretation of these $∖beta$ coefficients provides critical insights into the plant's operational dynamics. The dominant negative coefficient for B‐MLSS *β* = −0.4012 for e‐COD) aligns with the fundamental principles of the activated sludge process; it indicates that a higher concentration of active biomass in the aeration tank enhances the degradation of organic matter, directly resulting in lower effluent COD and SS levels. Biochemically, a higher concentration of active biomass provides a robust microbial population capable of synthesizing enzymes to rapidly oxidize and assimilate influent organic compounds, directly facilitating the degradation process and thereby lowering effluent COD and SS levels. Conversely, the positive coefficients observed for influent parameters such as i‐TN and i‐SS imply that shock loads in the influent exceed the instantaneous treatment capacity, leading to a proportional increase in effluent concentrations. Furthermore, the positive coefficient for temperature (B‐T) suggests that while biological activity increases with temperature, deviations beyond the optimal range or associated changes in oxygen solubility may negatively impact the settling properties of the sludge, leading to slightly higher effluent turbidity.

The variable with the most significant effect on the e‐COD output is observed to be B‐MLSS (*β* = −0.4012). This negative coefficient indicates that the B‐MLSS concentration reduces the e‐COD concentration at the outlet. Furthermore, variables such as *Q*, i‐pH, i‐TN, i‐TP, and B‐T have positive coefficients and offer limited but significant effects on e‐COD. B‐T (*β* = 0.0450) particularly emphasizes the effect of temperature change on e‐COD.

The highest effect on the e‐SS output was again observed in the B‐MLSS variable (*β* = −0.4127). This indicates that bioburden plays a critical role in determining e‐SS concentration. Furthermore, variables such as i‐SS, i‐TN, and B‐T contribute to the model with positive coefficients. i‐TN, in particular (*β* = 0.0224), demonstrates that nitrogen load has a direct effect on e‐SS. Additionally, higher influent nitrogen loads (i‐TN) can stimulate excessive microbial growth and increase the concentration of metabolic byproducts, which subsequently impacts the settling characteristics in the clarifier and mildly elevates the suspended solids and organic content in the effluent.

For e‐pH output, the most dominant variable is B‐MLSS (*β* = −0.3965). This negative relationship indicates that bioburden lowers e‐pH. Furthermore, variables such as i‐pH, i‐TN, i‐COD, and B‐T have positive coefficients, demonstrating that system inlet and biological pool parameters play a decisive role in e‐pH. B‐T (*β* = 0.0387), in particular, highlights the effect of temperature changes on pH balance.

In general, the B‐MLSS parameter stands out with high absolute *β* values for all output variables, indicating that the system's biological load is a dominant determinant of effluent water quality. These findings demonstrate that the model accurately represents both physical and chemical processes and has a strong predictive capacity.

Figure 4a–c shows how the cost function J(β) changes over time during the optimization process of the *β* parameters of the linear regression model created for e‐COD, e‐SS, and e‐pH outputs using the GDA. The horizontal axis represents the number of iterations, and the vertical axis represents the cost function value J(β).

The graph shows that the algorithm initially produces a rapid decrease in the cost function, then the decrease gradually slows down, and the function converges to a minimum value. Note that 10,000 iterations were run as the iteration. This typical trend demonstrates that the GDA operates effectively on a convex cost function and that the learning process has been successful. The initial steep slope indicates that the model parameters are initialized from a random or distant starting point, and the algorithm rapidly reduces cost by taking large steps in early iterations. The decreasing slope in later iterations indicates that the learning rate is appropriately chosen and the algorithm is steadily converging toward the minimum point.

These results demonstrate that the model's training process was successful and that the resulting *β* parameters provide high accuracy in representing the output of interest. Furthermore, the monotonically decreasing cost function implies that the model is free from problems such as overfitting or oscillation.

### 3.5 Graphical and numerical evaluation of model performance: Comparative analysis on e‐COD, e‐SS, and e‐pH outputs

**FIGURE 5:**
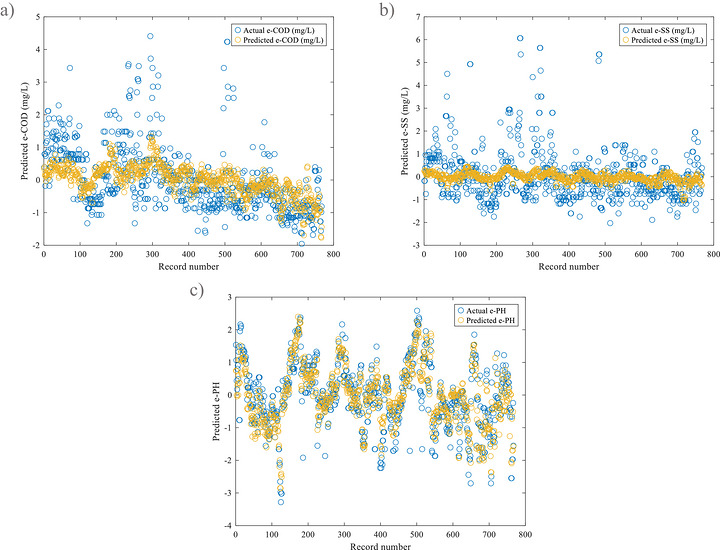
True‐predicted output values on test data: A regression model perspective. (a) Effluent chemical oxygen demand (e‐COD) output, (b) effluent suspended solids (e‐SS) output, and (c) effluent pH (e‐pH) output.

Figure 5a visually presents the performance of the e‐COD output predicted by the linear regression model on the test data. The graph shows a data‐based comparison of the actual (*y*) values with the model‐predicted (*ŷ*) values.

This comparison, presented through blue and yellow markers, shows that the model's predictions generally converge to the true values. The majority of the predicted values overlap highly with the true values, demonstrating that the model's accuracy is satisfactory. The low difference between the predictions and observations, particularly across a large portion of the dataset, demonstrates the model's strong generalization capacity. However, the presence of some local deviations indicates that the model may produce unsystematic errors in certain instances. These deviations may be due to variance in the dataset, measurement errors, or explanatory variables not included in the model. However, the general trend supports the model's ability to successfully represent the e‐COD output and that the linear regression approach is a valid method in this context.

Figure 5b visually presents the performance of the e‐SS output predicted by the linear regression model on the test data. The graph shows a data‐based comparison of the actual response values (*y*) with the model‐predicted values (*ŷ*). The blue markers in the graph represent the observed true values, while the yellow markers represent the model‐predicted values. The large overlap of the predicted values with the actual values indicates that the model successfully represents the e‐SS output. The low difference between the predictions and observations, particularly across a large portion of the dataset, demonstrates the model's high accuracy and strong generalization capacity. However, the presence of some local deviations suggests that the model may produce unsystematic errors in certain examples. These deviations may be due to variance in the dataset, measurement uncertainties, or explanatory variables not included in the model. However, the general trend supports the linear regression model as a valid and reliable predictor of e‐pH output.

Figure 5c shows the performance of the linear regression model's e‐pH output on test data. The graph compares the distribution of the model‐predicted values (*y*) with the actual response values (*ŷ*) based on the record number. Blue markers represent the observed actual e‐pH values, while yellow markers represent model predictions. The large overlap of the predicted values with the actual values demonstrates that the model successfully represents the e‐pH output. Predictions concentrated in the normalized range of −1 to +1, in particular, demonstrate that the system accurately models the pH balance and offers high accuracy for low‐variance outputs. The overall parallelism in the graph demonstrates that the model can capture fundamental pH dynamics and that the linear regression approach is a valid method in this context. However, deviations observed at some extreme values suggest that the model cannot fully represent extreme pH changes. This may be related to limitations of the linear model or inadequate explanatory variables in the dataset. These graphs are an important tool for assessing model reliability, both visually and statistically. Monitoring predictive performance over time or records, in particular, supports the model's usability in operational applications.

**FIGURE 6:**
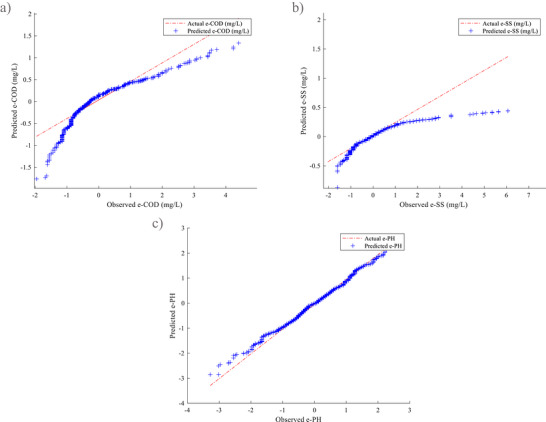
Prediction accuracy scatter plot for (a) effluent chemical oxygen demand (e‐COD) output, (b) effluent suspended solids (e‐SS) output, and (c) effluent pH (e‐pH) output.

Figure 6a presents the relationship between e‐COD values predicted by the linear regression model and observed (actual) values via a two‐dimensional scatter plot. The horizontal axis shows the actual *y* values, and the vertical axis shows the model's predicted (*y ^*) values, with the blue dots representing individual predictions. The red dashed line is the theoretical reference line representing the ideal prediction situation (the *y* = *x* line). In addition to assessing accuracy, these scatter plots serve as a visual analysis of prediction uncertainty. The tight clustering of data points around the reference line, with minimal dispersion particularly in the normalized range, indicates low residual variance and suggests that the model's error margins remain consistent across different operating conditions, rather than exhibiting heteroscedastic behavior

The majority of the blue dots in the graph are positioned close to the red reference line, indicating that the model achieved high accuracy in predictions. This convergence demonstrates that the predicted values do not contain systematic deviations and that the model successfully captures both mean trends and variance structure. However, some points deviating from the reference line indicate local prediction errors. These deviations suggest that the model's linear assumptions may be inadequate in certain data subsets or that the explanatory variables may not fully represent the effect of e‐COD in some samples. Overall, this scatter plot visually confirms the predictive performance of the linear regression model on e‐COD output and supports the model's statistical reliability.

The locations of the points in Figure 6b near the reference line indicate that the model generally successfully predicted e‐SS output. However, deviations, particularly observed at extreme values, suggest that the model's linear structure was insufficient in some examples and that the variance was not fully captured. The narrow range of the *y*‐axis (−1.5 to +1.5) indicates that the model's predictions are low‐variance and centered. This indicates that the model produces more conservative predictions against extremes and consistently represents the overall behavior of the system.

In Figure 6c, the red dashed line in the graph is the reference line representing the ideal prediction situation, and each blue dot represents the model's prediction on a test sample. The locations of the points close to this reference line and symmetrically indicate that the model predicted e‐pH output with high accuracy and consistency. Data points concentrated in the range of −2 to +2 demonstrate that the system's pH behavior is successfully represented by the linear regression model.

This visualization allows us to evaluate not only the prediction accuracy but also whether the model generates systematic errors. The even distribution of points around the reference line indicates that the model provides consistent results in terms of both bias and variance. This indicates that the model is free from classical regression problems such as overfitting or underfitting.

In conclusion, Figure 6 strikingly demonstrates the predictive performance of linear regression models on e‐COD, e‐SS, and e‐pH output, demonstrating strong modeling success in terms of both accuracy and dispersion. These graphs demonstrate that the model is reliable not only statistically but also at an operational level and can be used as an effective tool for predicting the balance of environmental systems. Such graphs are critical not only for the level of accuracy but also for analyzing the distribution structure of the predictions and potential areas of deviation. The RMSE, VAF, and $R_{\mathrm{adj}}^{2}$ values of the output parameters are displayed in Table 7.

**TABLE 7:** Meansquareerror(MSE), variance accounting factor (VAF), and $R_{\mathrm{adj}}^{2}$ values of the predicted values.

Outputs	RMSE		VAF		$R_{\mathrm{adj}}^{2}$	
	Train	Test	Train	Test	Train	Test
e‐COD	0.2965	0.2771	90.2344	91.9362	0.892	0.910
e‐SS	0.3009	0.2792	89.5956	92.2587	0.885	0.913
e‐pH	0.2784	0.3240	91.1857	88.6390	0.901	0.873

Abbreviations: e‐COD, effluent chemical oxygen demand; e‐pH, effluent pH; e‐SS, effluent suspended solids; RMSE, root mean square error; VAF, variance accounting factor.

The regression performance metrics presented in Table 7, based on normalized values, demonstrate that very high accuracy levels were achieved for e‐COD, e‐SS, and e‐pH outputs on both the training and test sets. In particular, the RMSE, VAF, and adjusted coefficient of determination *R* ^2^_adj_ values strongly demonstrate the generalization ability and explanatory power of the models.

1. The lowest RMSE (0.2771) and highest VAF (91.94%) obtained on the test set for the e‐COD output indicate that this parameter was most consistently estimated by the model. This is consistent with the low error distribution and high correlation structure observed in the previous graphs. The estimated *R* ^2^_adj_ value of 0.910 on the test set confirms the model's very high explanatory power.
2. The e‐SS output achieved the highest VAF value (92.26%) on the test set, demonstrating that the model consistently learned this parameter. The low RMSE value (0.2792) and the *R* ^2^_adj_ value reaching 0.913 are consistent with the homogeneous error distribution and linear fit in the previous graphs. These results demonstrate that the model developed for e‐SS has optimal performance in terms of both accuracy and generalization.
3. Although the e‐pH model achieved a high VAF of 91.19% during the training phase, this metric declined to 88.64% in the testing phase, consistent with the increased residual variance observed in the scatter plots. The corresponding rise in RMSE (0.3240) and the decrease in *R* ^2^_adj_ to 0.873 indicate that the model's generalization capability for e‐pH is slightly lower than for mass‐based parameters such as e‐COD and e‐SS. This disparity is primarily attributed to the fundamental definition of pH as a logarithmic function of hydrogen ion activity (pH = −log[H+]). Unlike mass‐concentration parameters, pH exhibits intrinsic nonlinear responses to influent variations, which a linear regression model can only approximate. While the current linear framework yields satisfactory results for operational monitoring, future implementations utilizing nonlinear architectures could further refine prediction accuracy by better capturing these logarithmic dependencies.

### 3.6 Practical evaluation of the results

Various approaches related to the actual plant have been presented as a result of preliminary investigations, data collection, data interpretation, and algorithm development to obtain results. In this context, once various plant parameters are measured, the target estimation parameter can be estimated using the model developed in this study. First, online sensors (such as COD, SS, pH, and temperature) will be placed at the sampling locations to define the data and will be transmitted simultaneously to the software via a network. This algorithm will allow for direct determination of effluent parameters. This process will likely increase energy consumption, particularly in the blower unit. By integrating the plants with existing supervisory control and data acquisition systems to manage energy consumption based on parameter estimations, plant users will have the opportunity to monitor the process in real time. This will improve plant infrastructure, and with remote connection and sensing systems, well‐managed plants can be operated more economically. Furthermore, improved effluent quality will reduce pollution released to the receiving environment.

## 4 CONCLUSIONS

Using the KOIZ‐WWTP raw wastewater parameters and some parameters from the biological treatment section of the plant, the effluent parameters e‐COD, e‐SS, and e‐pH were estimated using a ML‐based GDA model (batch gradient descent variant) using a linear regression model. This two‐stage study first determined the relationship between 11 parameters and the output parameters by applying correlation selection to the input parameters. A median filter was applied to the dataset used in the study with the parameters with the highest correlation. This way, potential outliers were reduced to smoother values, reducing noise in the data. After applying the model to the training dataset, the study was conducted on the test dataset. As a result, RMSE values of e‐COD, e‐SS, and e‐pH were obtained as 0.2771, 0.2792, and 0.3240, respectively. Furthermore, the VAF values were calculated as 91.9362, 92.2587, and 88.6390. Finally, 0.910, 0.913, and 0.873 were calculated for the *R* ^2^_adj_ value. Accordingly, the model was accurately determined iteratively without overfitting. This study aims to reduce laboratory analysis, operation, maintenance, labor, and time costs as a result of the estimation of the plant's discharge water parameters. The robust predictive success achieved by this linear regression framework demonstrates that the core dynamics of the KOIZ‐WWTP can be effectively modeled with high transparency. This achievement serves as a vital proof‐of‐concept, paving the way for more advanced modeling strategies. Future research will focus on implementing sophisticated time‐series algorithms, such as long short‐term memory networks and nonlinear autoregressive exogenous models. These architectures are expected to further refine prediction accuracy by capturing the potential long‐term temporal dependencies and nonlinearities inherent in wastewater characteristics over extended operational periods.

## AUTHOR CONTRIBUTIONS

**Mahmut Sami Sasmazturk**: Conceptualization; formal analysis; methodology; writing—original draft; writing—review and editing. **Alper Solmaz**: Conceptualization; investigation; supervision; validation; writing—original draft; writing—review and editing. **Talip Turna**: Conceptualization; data curation; investigation; supervision; writing—original draft; writing—review and editing.

## CONFLICT OF INTEREST STATEMENT

The authors declare no conflicts of interest.

## Data Availability

The datasets generated during and/or analyzed during the current study are available from the corresponding author on reasonable request.

## References

[jeq270204-bib-0001] Alpaydin, E. (2020). Introduction to machine learning (4th ed.). The MIT Press.

[jeq270204-bib-0002] Asuero, A. G. , Sayago, A. , & González, A. G. (2006). The correlation coefficient: An overview. Critical Reviews in Analytical Chemistry, 36(1), 41–59. 10.1080/10408340500526766

[jeq270204-bib-0003] Bagherzadeh, F. , Mehrani, M. J. , Basirifard, M. , & Roostaei, J. (2021). Comparative study on total nitrogen prediction in wastewater treatment plant and effect of various feature selection methods on machine learning algorithms performance. Journal of Water Process Engineering, 41, 102033. 10.1016/J.JWPE.2021.102033

[jeq270204-bib-0004] Bashir Shaban, K. , Kadri, A. , & Rezk, E. (2016). Urban air pollution monitoring system with forecasting models. IEEE Sensors Journal, 16(8), 2598–2606. 10.1109/JSEN.2016.2514378

[jeq270204-bib-0005] Borzooei, S. , Campo, G. , Cerutti, A. , Meucci, L. , Panepinto, D. , Ravina, M. , Riggio, V. , Ruffino, B. , Scibilia, G. , & Zanetti, M. (2019). Optimization of the wastewater treatment plant: From energy saving to environmental impact mitigation. Science of the Total Environment, 691, 1182–1189. 10.1016/J.SCITOTENV.2019.07.241 31466200

[jeq270204-bib-0006] Chen, Y. , Song, L. , Liu, Y. , Yang, L. , & Li, D. (2020). A review of the artificial neural network models for water quality prediction. Applied Sciences, 10(17), 5776. 10.3390/APP10175776

[jeq270204-bib-0007] Cheng, Q. , Chunhong, Z. , & Qianglin, L. (2023). Development and application of random forest regression soft sensor model for treating domestic wastewater in a sequencing batch reactor. Scientific Reports, 13(1), 9149. 10.1038/s41598-023-36333-8 37277429 PMC10241833

[jeq270204-bib-0008] Ching, P. M. L. , So, R. H. Y. , & Morck, T. (2021). Advances in soft sensors for wastewater treatment plants: A systematic review. Journal of Water Process Engineering, 44, 102367. 10.1016/J.JWPE.2021.102367

[jeq270204-bib-0009] Desboulets, L. D. D. (2018). A review on variable selection in regression. Econometrics, 6(4), 45. 10.3390/econometrics6040045

[jeq270204-bib-0010] Gabbouj, M. , Coyle, E. J. , & Gallagher, N. C. (1992). An overview of median and stack filtering. Circuits, Systems and Signal Processing, 11(1), 7–45. 10.1007/BF01189220

[jeq270204-bib-0011] Géron, A. (2019). Hands‐on machine learning with Scikit‐Learn, Keras, and TensorFlow : Concepts, tools, and techniques to build intelligent systems. O'Reilly Media Inc..

[jeq270204-bib-0012] Geweke, J. (1996). Variable selection and model comparison in regression (Working Papers 539). Federal Reserve Bank of Minneapolis. https://www.researchgate.net/publication/2351555

[jeq270204-bib-0013] Guo, H. , Jeong, K. , Lim, J. , Jo, J. , Kim, Y. M. , Park, J.‐P. , Kim, J. H. , & Cho, K. H. (2015). Prediction of effluent concentration in a wastewater treatment plant using machine learning models. Journal of Environmental Sciences, 32, 90–101. 10.1016/J.JES.2015.01.007 26040735

[jeq270204-bib-0014] Haimi, H. , Mulas, M. , Corona, F. , & Vahala, R. (2013). Data‐derived soft‐sensors for biological wastewater treatment plants: An overview. Environmental Modelling & Software, 47, 88–107. 10.1016/J.ENVSOFT.2013.05.009

[jeq270204-bib-0015] Haji, S. H. , & Abdulazeez, A. M. (2021). Comparison of optimization techniques based on gradient descent algorithm: A review. PalArch's Journal of Archaeology of Egypt/Egyptology, 18(4), 2715–2743.

[jeq270204-bib-0016] Hamza, R. A. , Zaghloul, M. S. , Iorhemen, O. T. , Sheng, Z. , & Tay, J. H. (2019). Optimization of organics to nutrients (COD:N:P) ratio for aerobic granular sludge treating high‐strength organic wastewater. Science of the Total Environment, 650, 3168–3179. 10.1016/J.SCITOTENV.2018.10.026 30373093

[jeq270204-bib-0017] Hastie, T. , Tibshirani, R. , Friedman, J. , & Franklin, J. (2005). The elements of statistical learning: Data mining, inference and prediction. The Mathematical Intelligencer, 27(2), 83–85.

[jeq270204-bib-0018] Huang, L. (2022). Normalization techniques in deep learning (1st ed.) Springer International Publishing.

[jeq270204-bib-0019] Huang, R. , Ma, C. , Ma, J. , Huangfu, X. , & He, Q. (2021). Machine learning in natural and engineered water systems. Water Research, 205, 117666. 10.1016/J.WATRES.2021.117666 34560616

[jeq270204-bib-0020] Hvala, N. , & Kocijan, J. (2020). Design of a hybrid mechanistic/Gaussian process model to predict full‐scale wastewater treatment plant effluent. Computers & Chemical Engineering, 140, 106934. 10.1016/j.compchemeng.2020.106934

[jeq270204-bib-0021] Jafar, R. , Awad, A. , Jafar, K. , & Shahrour, I. (2022). Predicting effluent quality in full‐scale wastewater treatment plants using shallow and deep artificial neural networks. Sustainability, 14(23), 15598. 10.3390/su142315598

[jeq270204-bib-0022] Jana, D. K. , Bhunia, P. , Das Adhikary, S. , & Bej, B. (2022). Optimization of effluents using artificial neural network and support vector regression in detergent industrial wastewater treatment. Cleaner Chemical Engineering, 3, 100039. 10.1016/j.clce.2022.100039

[jeq270204-bib-0023] Khasnabis, A. , Parthasarathy, C. , Rani, A. , Kokurde, V. R. , & Subbiah, S. (2025). Assessment of the condition of a wastewater treatment plant. In S. Souabi , A. Anouzla , S. Yadav , V. P. Singh , & R. N. Yadava (Eds.), Wastewater treatment plants—Processes, assessment, design and operation (pp. 215–267). Springer.

[jeq270204-bib-0024] Lecun, Y. , Bengio, Y. , & Hinton, G. (2015). Deep learning. Nature, 521(7553), 436–444. 10.1038/nature14539 26017442

[jeq270204-bib-0025] Lee, B. C. Y. , Mahtab, M. S. , Neo, T. H. , Farooqi, I. H. , & Khursheed, A. (2022). A comprehensive review of design of experiment (DOE) for water and wastewater treatment application—Key concepts, methodology and contextualized application. Journal of Water Process Engineering, 47, 102673. 10.1016/J.JWPE.2022.102673

[jeq270204-bib-0026] Makwana, D. , Engineer, P. , Dabhi, A. , & Chudasama, H. (2023). Sampling methods in research: A review. International Journal of Trend in Scientific Research and Development, 7(3), 762–768.

[jeq270204-bib-0027] Martin, R. , & Lutz, R. K. (1979). Background following on Schmidt plates using a digital filtering technique. In International Workshop on Image Processing in Astronomy (pp. 211–217). Osservatorio Astronomico di Trieste.

[jeq270204-bib-0028] Martino, L. , Luengo, D. , & Míguez, J. (2018). Independent random sampling methods. Springer International Publishing.

[jeq270204-bib-0029] Metcalf & Eddy . (2013). Wastewater engineering: Treatment and resource recovery (5th ed.) McGraw‐Hill Education.

[jeq270204-bib-0030] Nawaz, A. , Arora, A. S. , Ali, W. , Saxena, N. , Khan, M. S. , Yun, C. M. , & Lee, M. (2022). Intelligent human–machine interface: An agile operation and decision support for an ANAMMOX SBR system at a pilot‐scale wastewater treatment plant. IEEE Transactions on Industrial Informatics, 18(9), 6224–6232. 10.1109/TII.2022.3153468

[jeq270204-bib-0031] Noori, R. , Berndtsson, R. , Hosseinzadeh, M. , Adamowski, J. F. , & Abyaneh, M. R. (2019). A critical review on the application of the National Sanitation Foundation Water Quality Index. Environmental Pollution, 244, 575–587. 10.1016/J.ENVPOL.2018.10.076 30384063

[jeq270204-bib-0032] Pisa, I. , Santín, I. , Vicario, J. L. , Morell, A. , & Vilanova, R. (2019). ANN‐based soft sensor to predict effluent violations in wastewater treatment plants. Sensors, 19(6), 1280. 10.3390/S19061280 30871281 PMC6470776

[jeq270204-bib-0033] Plevris, V. , Solorzano, G. , Bakas, N. , & Seghier, M. B (2022). Investigation of performance metrics in regression analysis and machine learning‐based prediction models. In 8th European Congress on Computational Methods in Applied Sciences and Engineering (pp. 1–25). CIMNE.

[jeq270204-bib-0034] Pratt, W. K. (2013). Introduction to digital image processing. CRC press.

[jeq270204-bib-0035] Raposo, F. , Delarubia, M. , Borja, R. , & Alaiz, M. (2008). Assessment of a modified and optimised method for determining chemical oxygen demand of solid substrates and solutions with high suspended solid content. Talanta, 76(2), 448–453. 10.1016/J.TALANTA.2008.03.030 18585304

[jeq270204-bib-0036] Ruder, S. (2016). An overview of gradient descent optimization algorithms. *arXiv*. https://arxiv.org/pdf/1609.04747

[jeq270204-bib-0037] Salgot, M. , & Folch, M. (2018). Wastewater treatment and water reuse. Current Opinion in Environmental Science & Health, 2, 64–74. 10.1016/J.COESH.2018.03.005

[jeq270204-bib-0038] Shao, S. , Fu, D. , Yang, T. , Mu, H. , Gao, Q. , & Zhang, Y. (2023). Analysis of machine learning models for wastewater treatment plant sludge output prediction. Sustainability, 15(18), 13380. 10.3390/su151813380

[jeq270204-bib-0039] Sweeney, M. , & Kabouris, J. (2020). Modeling, instrumentation, automation, and optimization of water resource recovery facilities (2019) DIRECT. Water Environment Research, 92(10), 1499–1503. 10.1002/WER.1394 32639061

[jeq270204-bib-0040] Taylor, R. (1990). Interpretation of the correlation coefficient: A basic review. Journal of Diagnostic Medical Sonography, 6(1), 35–39. 10.1177/875647939000600106

[jeq270204-bib-0041] Teng, Y. , Wu, J. , Lu, S. , Wang, Y. , Jiao, X. , & Song, L. (2014). Soil and soil environmental quality monitoring in China: A review. Environment International, 69, 177–199. 10.1016/J.ENVINT.2014.04.014 24875802

[jeq270204-bib-0042] Tukey, J. W. (1977). Exploratory data analysis. Springer.

[jeq270204-bib-0043] Vanrolleghem, P. A. , Khalil, M. , Serrao, M. , Sparks, J. , & Therrien, J.‐D. (2025). Machine learning in wastewater: Opportunities and challenges—“not everything is a nail!”. Current Opinion in Biotechnology, 93, 103271. 10.1016/j.copbio.2025.103271 39999506

[jeq270204-bib-0044] Wan, X. , Li, X. , Wang, X. , Yi, X. , Zhao, Y. , He, X. , Wu, R. , & Huang, M. (2022). Water quality prediction model using Gaussian process regression based on deep learning for carbon neutrality in papermaking wastewater treatment system. Environmental Research, 211, 112942. 10.1016/j.envres.2022.112942 35189104

[jeq270204-bib-0045] Wang, D. , Thunéll, S. , Lindberg, U. , Jiang, L. , Trygg, J. , Tysklind, M. , & Souihi, N. (2021). A machine learning framework to improve effluent quality control in wastewater treatment plants. Science of the Total Environment, 784, 147138. 10.1016/J.SCITOTENV.2021.147138 34088065

[jeq270204-bib-0046] Wang, F. , Wang, Y. , Zhang, K. , Hu, M. , Weng, Q. , & Zhang, H. (2021). Spatial heterogeneity modeling of water quality based on random forest regression and model interpretation. Environmental Research, 202, 111660. 10.1016/j.envres.2021.111660 34265353

[jeq270204-bib-0047] Wang, F. , Zhu, J. , Chen, L. , Zuo, Y. , Hu, X. , & Yang, Y. (2020). Autonomous and in situ ocean environmental monitoring on optofluidic platform. Micromachines, 11(1), 69. 10.3390/MI11010069 31936398 PMC7019421

[jeq270204-bib-0048] Wang, X. , Yan, L. , & Zhang, Q. (2021). Research on the application of gradient descent algorithm in machine learning. In 2021 international conference on computer network, electronic and automation (pp. 11–15). ICCNEA. 10.1109/ICCNEA53019.2021.00014

[jeq270204-bib-0049] Wen, H. , Lee, C. C. , & Song, Z. (2021). Digitalization and environment: How does ICT affect enterprise environmental performance? Environmental Science and Pollution Research, 28(39), 54826–54841. 10.1007/S11356-021-14474-5 34014481

[jeq270204-bib-0050] Ye, F. , Ye, Y. , & Li, Y. (2011). Effect of C/N ratio on extracellular polymeric substances (EPS) and physicochemical properties of activated sludge flocs. Journal of Hazardous Materials, 188(1–3), 37–43. 10.1016/J.JHAZMAT.2011.01.043 21333444

[jeq270204-bib-0051] Ye, Z. , Yang, J. , Zhong, N. , Tu, X. , Jia, J. , & Wang, J. (2020). Tackling environmental challenges in pollution controls using artificial intelligence: A review. Science of the Total Environment, 699, 134279. 10.1016/J.SCITOTENV.2019.134279 33736193

[jeq270204-bib-0052] Yegnanarayana, B. (2009). Artificial neural networks. PHI Learning Pvt. Ltd.

[jeq270204-bib-0053] Zhang, W. , Tooker, N. B. , & Mueller, A. V. (2020). Enabling wastewater treatment process automation: Leveraging innovations in real‐time sensing, data analysis, and online controls. Environmental Science: Water Research & Technology, 6(11), 2973–2992. 10.1039/D0EW00394H

[jeq270204-bib-0054] Zolkefli, N. , Sharuddin, S. S. , Yusoff, M. Z. M. , Hassan, M. A. , Maeda, T. , & Ramli, N. (2020). A review of current and emerging approaches for water pollution monitoring. Water, 12(12), 3417. 10.3390/W12123417

